# Entrustable professional activities (EPAs) for undergraduate medical education – development and exploration of social validity

**DOI:** 10.1186/s12909-023-04621-6

**Published:** 2023-09-04

**Authors:** Christina Gummesson, Stina Alm, Anna Cederborg, Mattias Ekstedt, Jarl Hellman, Hans Hjelmqvist, Magnus Hultin, Katarina Jood, Charlotte Leanderson, Bertil Lindahl, Riitta Möller, Björn Rosengren, Anders Själander, Peter J Svensson, Stefan Särnblad, Alexander Tejera

**Affiliations:** 1 Faculty of Medicine, Faculty of Odontology, Lund University, Malmö University, Malmö, Sweden; 2 Department of Clinical Sciences, Futurum - the Academy for Health and Care, Region Jönköping County, Umeå University, Paediatrics, Umeå, Sweden; 3 Institute of Medicine, Sahlgrenska Academy, Department of Medicine, University of Gothenburg, Sahlgrenska University Hospital, Gothenburg, Sweden; 4 Department of Health, Medicine and Caring Sciences, Linköping University, Linköping, 581 83 Sweden; 5 Department of Medical Sciences, Uppsala University, Uppsala, Sweden; 6 Department of Anesthesiology and Intensive Care, Faculty of Medicine and Health, Örebro University, Örebro, Sweden; 7 Department of Surgical and Perioperative Sciences, Anesthesiology and Intensive Care Medicine, Umeå University, Umeå, Sweden; 8 Department of Clinical Neuroscience, Institute of Neuroscience and Physiology, Sahlgrenska Academy, Department of Neurology, University of Gothenburg, Sahlgrenska University Hospital, Gothenburg, Sweden; 9 Department of Neurobiology, Care Sciences and Society, Karolinska Institutet, Stockholm, Sweden; 10 Department of Clinical Science, Intervention and Technology, Department of Medical Epidemiology and Biostatistics, Karolinska Institutet, Karolinska Institutet, Stockholm, Sweden; 11 Clinical and Molecular Osteoporosis Research Unit, Department of Orthopedics and Clinical Sciences, Skåne University Hospital Malmö, Lund University, Malmö, Sweden; 12 Department of Public Health and Clinical Medicine, Umeå University, Umeå, Sweden; 13 Department of Clinical Sciences Lund University, Skåne University Hospital, Malmö, Sweden; 14 Department of Pediatrics, Faculty of Medicine and Health, Örebro University, Örebro, Sweden; 15 Division of Translational Cancer Research, Department of Laboratory Medicine, Skåne University Hospital, Lund University, Lund University, Lund, Sweden

**Keywords:** Entrustable professional activities, Development, Social validity

## Abstract

**Background:**

The development of entrustable professional activities (EPAs) as a framework for work-based training and assessment in undergraduate medical education has become popular. EPAs are defined as units of a professional activity requiring adequate knowledge, skills, and attitudes, with a recognized output of professional labor, independently executable within a time frame, observable and measurable in its process and outcome, and reflecting one or more competencies. Before a new framework is implemented in a specific context, it is valuable to explore social validity, that is, the acceptability by relevant stakeholders.

**Aim:**

The aim of our work was to define Core EPAs for undergraduate medical education and further explore the social validity of the constructs.

**Method and material:**

In a nationwide collaboration, EPAs were developed using a modified Delphi procedure and validated according to EQual by a group consisting of teachers nominated from each of the seven Swedish medical schools, two student representatives, and an educational developer (n = 16). In the next step, social validity was explored in a nationwide survey. The survey introduced the suggested EPAs. For each EPA, the importance of the EPA was rated, as was the rater’s perception of the present graduates’ required level of supervision when performing the activity. Free-text comments were also included and analyzed.

**Results:**

Ten Core EPAs were defined and validated. The validation scores for EQual ranged from 4.1 to 4.9. The nationwide survey had 473 responders. All activities were rated as “important” by most responders, ranging from 54 to 96%. When asked how independent current graduates were in performing the ten activities, 6 to 35% reported “independent”. The three themes of the free text comments were: ‘relevant target areas and content’; ‘definition of the activities’; and ‘clinical practice and learning’.

**Conclusion:**

Ten Core EPAs were defined and assessed as relevant for Swedish undergraduate medical education. There was a consistent gap between the perceived importance and the certainty that the students could perform these professional activities independently at the time of graduation. These results indicate that the ten EPAs may have a role in undergraduate education by creating clarity for all stakeholders.

## Background

A global as well as local challenge is to describe and communicate what newly graduated medical students should be able to perform and, thereby, what they need to practice during their education. Extensive work is being done to describe the knowledge and skills that medical students should have by the time they graduate. Given the large number of teachers and supervisors at each medical school, creating descriptions that can be easily understood and interpreted and thus used during education is a challenge. The use of competencies as descriptors for medical education is one way of describing the complex integration of knowledge, skills, and behavior required. Competency-based education can be defined in a variety of ways. An international definition of a competence based education includes five components: outcome competencies, sequenced progression, tailored learning experiences, competency-focused instruction, and programmatic assessment [1]. Our focus in this paper is on outcomes. Promoted in Europe by the Bologna Process [2, 3] these are frequently articulated as knowledge, skills, and attitudes.

The end goal of the descriptions is to clarify the requirements for the students’ performance at the time of graduation. The students’ performance should then be at a level where they engage to improve patient care and meet the health care needs of the population [4]. For descriptions to serve that purpose, they should ideally be defined and expressed in a way that is concise enough to grasp, and accepted by teachers, supervisors, and students. In our experience, competencies and intended learning outcomes may not be easily communicated and interpreted among the different stakeholders. It has also been proposed that the application of competency descriptions in training may detract attention from the students’ performance on actual patient care [5] due, for example, to the challenge of translating competency domains into patient care where several competencies are integrated [6].

Entrustable Professional Activities (EPAs), a concept developed by ten Cate [7], has become widely used as a framework for training and assessment of clinical activities. A recent overall definition of an EPA is ‘a unit of professional practice that can be fully entrusted to a trainee, once he or she has demonstrated the necessary competence to execute this activity unsupervised’ [8]. The activity should be limited to work-based professional practice, be observable, be limited in time, and have a clearly defined beginning and end so that the level of supervision needed can be assessed and used for feedback throughout the education. From the beginning, the concept was used for specialist training [9], however the model has since been refined [10, 11] and proposed to be useful for undergraduate education as well [5]. The framework has gained increasing popularity internationally, also at the undergraduate level, with published frameworks from Australia, Canada, Germany, Mexico, the Netherlands, Switzerland, and the United States [12].

An argument for defining undergraduate EPAs is thus to clarify which professional activities need to be purposefully and systematically trained and mastered, with a specified level of supervision, during undergraduate education. The expected degree of autonomy and under what circumstances the student should be able to perform each activity should be agreed upon nationally for the undergraduate level, as some activities may still be at a level requiring supervision but are important in preparation for independence at post-graduate training. EPAs have the potential to be a manageable framework for frequent training, assessment, and feedback during undergraduate education.

Although EPAs constitute also a complex framework to develop, we believe that expressions of activities may be more easily understood and utilized by the different stakeholders than competencies. The exact phrasing of EPAs is recognized to be difficult and sometimes confused with other frameworks used within medical education [13], and the use of a systematic process is recommended for the development of EPAs [14].

The Delphi method is an approach to structuring communication and developing consensus that is widely used in medical education research, including the development of EPA [10, 15, 16]. It consists of an iterative process with several stages, starting with the identification of the research problem, selection of participants, search of the literature, and development of a questionnaire with statements, followed by the deployment of anonymous iterative questionnaire rounds among the participants. For each round, the researcher feeds back the results of the previous round. The iterative process is repeated until the best possible level of consensus is reached, or a predetermined number of rounds are completed.

As the EPA framework is intended to be used by many supervisors and its acceptance is important, aspects of the validity of the construct need to be assessed as proposed in the framework on social validity by Wolf [17] and the validity framework by Messick [18], addressing the concerns of the social dimension, e.g., by questioning the social and cultural assumptions underlying the construct. Social validity concerns the perceived acceptability and importance of a test or outcome among target populations [19] and involves the stakeholders’ agreement on the significance of the goals, appropriateness, and importance of the outcomes [17]. The importance of using a mixed-method approach to gather information was recently suggested in a scoping review on social validity in clinical research [19].

The aim of our work was to define Core EPAs for undergraduate medical education and further explore the social validity of the constructs.

## Method

### Study design

The development of nationwide undergraduate EPAs was based on a socio-constructivist paradigm to inform our participatory design, methodology, and analysis. This meant that the development was socially situated close to the context where it was intended to be used, and the EPAs were constructed through social interaction within the group and with external stakeholders. The expertise in the group on medical education (all having extensive experience as supervisors and assessors) was capitalized on for a continuous reflexive dialogue, challenging definitions, and shared interpretations by contrasting the work to the current educational situation and the participants’ view of their own (historical) independence at the time of graduation. This also addressed contextual reflexivity and social acceptability by putting definitions into perspectives of the future, historical context, and current context within the research group and with external stakeholders. Altogether, a mixed methods approach seemed purposeful and has been recommended [20]. To capture qualitative aspects and understanding of concepts, yet to be able to capture an overall perception among a large group of external stakeholders, a quantitative approach was found suitable, accompanied by qualitative free text comments for clarification.

### Context

Undergraduate medical education leading to an MD degree is available at seven universities in Sweden. Each is operating autonomously. However, all medical educations are required to meet the specified national qualification requirements with defined knowledge, skills, and attitudes that students should possess upon graduation. The curriculum comprised 5.5 years but was about to change to 6 full years in fall 2021, i.e., when this project was completed. The major difference with the change was that the 6-year program, grounded in the new national requirements, would allow students to apply for a license without any further request for licensing assessment or practice. All medical education is built on exams at the course level only. This means that students need to progress in autonomy in relation to the supervisors during their clinical rotations (courses) to reach the final national outcomes, e.g., “demonstrate specialized skills in autonomously diagnosing the most frequent illnesses from pathophysiological and psychosocial, as well as other relevant perspectives, and in treating them in collaboration with the patients”.

The new national requirements were framed broadly, like the previous requirements, and include skills such as being able to independently integrate and use knowledge, diagnose diseases, and initiate treatment of life-threatening conditions. Clinical rotations and clinical activity training may be introduced during the first semesters at the pace and extent determined by each school. Throughout the clinical rotations, the students are supervised by medical doctors working at the university hospitals or hospitals and primary care centers affiliated with the university.

### Participants

The national expert group working on the development of EPAs consisted of two medical teachers nominated from each of the seven medical schools, two student representatives, and one educational developer. Due to unforeseen circumstances, one school only had one member participate in the whole process. Thus, in total, 16 participants took part in the whole process. The medical doctors/teachers were program leaders or clinical faculty responsible for rotations and assessments of students. The group represented various medical specialty fields (such as anesthesiology, cardiology, endocrinology, family medicine, internal medicine, neurology, orthopedic surgery, and pediatrics). The national organization for medical students nominated two students with previous experience in educational development.

The group (i.e., the authors of this paper) shared a strong desire to develop a purposeful framework that would enable assurance of graduates’ competencies. The project was endorsed by all medical schools and government stakeholders. The process described here was conducted from October 2018 until October 2019.

### The modified delphi approach

**Fig. 1:**
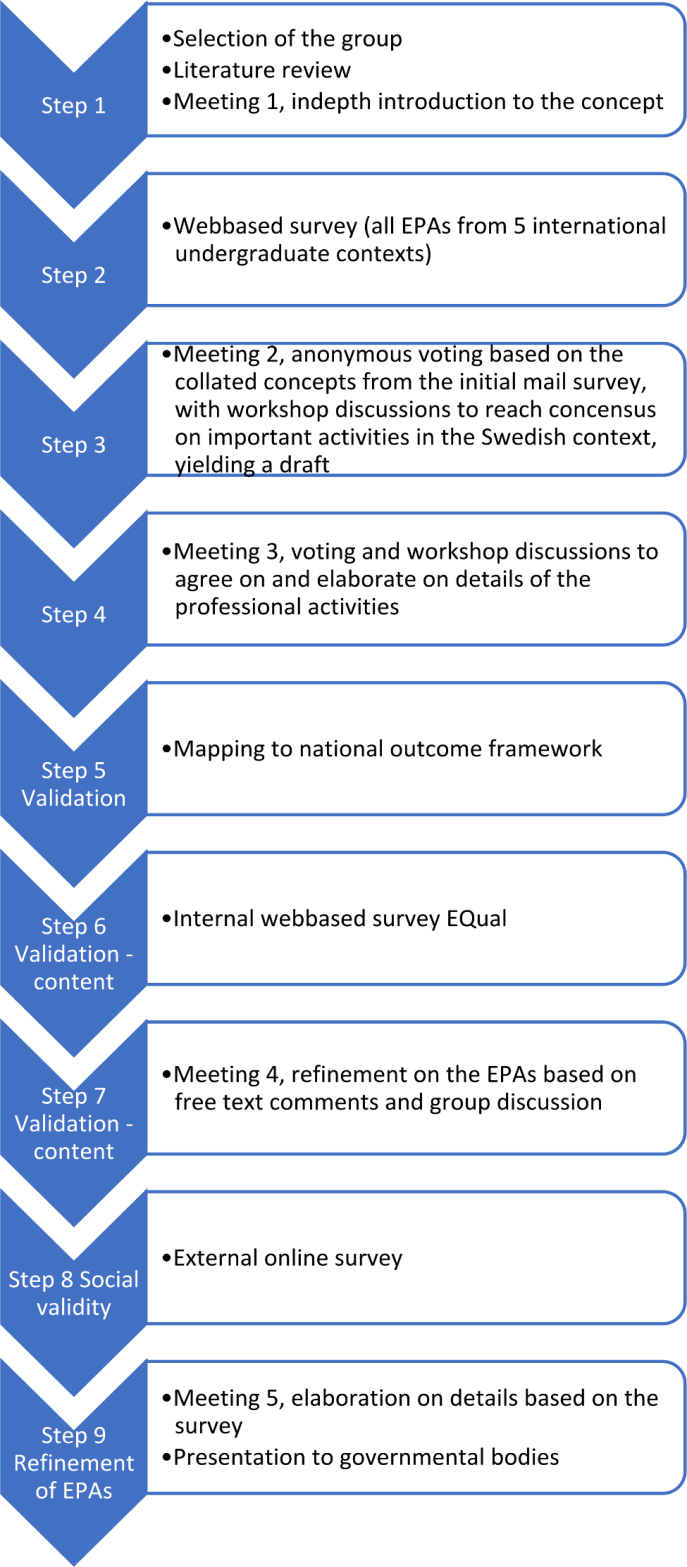
A description of the steps in the process of developing and initial validation of the undergraduate Core EPAs

Our modified Delphi approach consisted of four steps (Fig. 1). The developmental process was facilitated by the educational developer to first expand a shared understanding of the EPA concept within the group to prepare for the decision-making process. The development of the EPA framework was approached in a systematic and collaborative way to create consensus.

In the first step, the educational developer reviewed the literature for published undergraduate medical education EPAs. A first meeting was held, and the participants were comprehensively introduced to the concept of EPA. This step was followed by the first questionnaire round, which consisted of an anonymous online survey including EPAs from other undergraduate contexts identified from the literature review [9, 21–24]. In the survey, the participants were asked to grade each EPA according to its relevance to the Swedish context. The survey also included open-ended questions asking the participants if they missed any key professional activities in the survey. The results were summarized and sent to the participants before the second round. At the second and third meetings, anonymous polls were used iteratively with workshops where the participants worked in small groups to suggest elaborate formulations, followed by poll rounds to reach consensus. During the third meeting, details of the descriptions were elaborated on, and the expected level of autonomy was discussed.

Throughout the process, the theoretical concept was reviewed and revisited. This was done to ensure we would stay close to the original framework principles. The process was open in the sense that participants could talk with others between meetings to stay in touch with the local needs, but voting was done anonymously to avoid internal pressure. The process was then expanded to include aspects of validity and social acceptability in a wider context.

### Validity aspects

Our early validity exploration consisted of several steps (Fig. 1). First, the proposed EPAs were internally validated through an anonymous survey after the third round using the EPA Quality (EQual) measuring scoring rubric [11] to guide further revision. For each EPA, the 14 rubrics (representing 3 domains: discrete units of work = 6 items; entrustable, essential, and important tasks of the profession = 4 items; curricular role = 4 items) were scored, with the option of also adding free text comments. Each rubric was scored from 1 to 5, where 5 represents fulfilling the criteria. The mean score of the 14 rubrics was calculated for each EPA, for each responder and for the group. A cutoff group mean score of 4.07 has been proposed to identify substandard EPAs not meeting these requirements, thus needing revision [11]. As a post-hoc analysis, we also calculated the mean scores for each domain [25].

The second step of the validity exploration addressed social validity by contrasting the content with the national requirements and internally discussing the estimated level of autonomy at the time of graduation for current students and oneself. The EPAs were vetted against the new national outcome descriptors for medical doctors at their graduation from university as part of their content validity. This step was also intended to address the question of whether the EPAs addressed the government-mandated constructs. At the beginning and end of the process, national stakeholders were invited for a dialogue about the initiation of the project and the reporting of the results.

Finally, social validity was addressed by approaching medical doctors working with students and newly graduates through an online survey. Each EPA was presented with a description and limitations. The survey was developed with two questions repeated for each EPA. First, the perceived importance of the professional activity was rated on a 3-level rating scale from ‘not important’ to ‘important’. Second, the rater’s perception of the present graduates’ level of autonomy when performing the activity was recorded on a 3-level rating scale: ‘not at all’, ‘require supervision’, ‘independent’. Free text comments were allowed for each question, as well as a final question asking if the respondent thought there were other observable, common, and important activities that should be addressed. The intent was to make it possible to express thoughts about our construct in a survey designed as simply as possible to reduce the complexity and time required to respond.

This nationwide online survey was administered via the program chairs at each medical school. The responders were, in the introduction to the survey, informed about the purpose also being intended for research and scientific publications. Consent was required before entering the actual survey. The responses were collected anonymously, without any personal information being recorded. The only demographic information collected was years of clinical practice (< 5, 5–10, and > 10 years) and medical specialty.

### Analysis

Data from EQual and the nationwide survey were presented using descriptive statistics. We also wanted to investigate possible differences in opinion based on time in the profession from the standpoint of social validity. A post-hoc sub-analysis of responses from the survey was therefore performed for those who reported having been in the profession for ≤ 10 years or > 10 years. The chi-square test was used for the analysis of differences between the groups.

Data from the open-ended questions of the survey were used for the final revision and to present the content here, also analyzed by inductive qualitative content analysis according to the steps described by Graneheim and Lundman [26], including repeated reading, dividing the text into meaning units, then condensed to a manifest description, which was organized into sub-themes, and finally the themes were described. The written comments were read by two of the authors (CL and RM who both had extensive experience in content analysis research), who identified the meaning units and coded the text. The analysis process was then discussed with other members of the research group using an iterative process until consensus was reached about themes and sub-themes [26].

### Ethical considerations

The study followed the Helsinki declaration. Before accessing the survey, participants were informed about the purpose of the questionnaire and that the results could be used for scientific purposes and that by submitting their responses they agreed to the use and thereby gave their informed consent. No personal or sensitive information was collected. According to the Swedish Ethical Review Act, (SFS 2003:460), this kind of study is not subject to ethical review.

## Results

### Defining swedish EPAs and the level of supervision and entrustment

The stepwise process yielded 10 Core EPAs for Swedish undergraduate education (Table 1). An observational, retrospective, Entrustment-Supervision rating scale in Swedish was developed through research group consensus (Table 2). It was agreed on which EPAs should be at the level of autonomy from the supervisor at the time of graduation (i.e., the time for licensing) and which EPAs could only be ensured that all students would practice in a simulated environment (such as ‘Recognize patients requiring urgent care and initiate primary intervention’). Although, in these cases, students should reach a level where no prompts or supervisory intervention is required in the simulated environment.

**Table 1:** The Swedish Core EPAs for undergraduate education

1	Gather a history and perform a relevant physical examination
2	Prioritize a preliminary diagnosis among relevant differential diagnoses
3	Formulate an initial plan for investigations
4	Formulate and implement an initial management plan
5	Identify the need for and initiate interventions to promote health and prevent illness
6	Perform general procedures of a physician
7	Recognize patients requiring urgent care and initiate primary intervention
8	Summarize, document, prescribe and issue medical certificate based on a patient encounter
9	Collaborate within healthcare and with other professionals in the community
10	Contribute to a patient safety culture within healthcare

**Table 2:** A translation of the Swedish observation rating scale for undergraduate education. The scale is used together with an open-ended question for feedforward

Description
The student was an active observer while I did the activity
The student did the activity together with me
The student did the activity, I was there and had to intervene or prompt
The student did the activity, I was there and did not need to intervene or prompt
The student did the activity, I was nearby and had to intervene or prompt
The student did the activity, I was nearby and did not need to intervene or prompt

### Validation process

Once there was agreement on moving forward with the 10 EPA descriptions, the first part of the validation process was done within the group. Questions were posed to the participants about definitions and shared interpretations by contrasting the work with the current educational situation, including the participants’ experiences as assessors and supervisors, and the participants’ view of their own (historical) independence at the time of graduation. There was an agreement that, in the present education system, it was unclear to what extent the students were independent in performing any of the proposed activities. There was also agreement that previous and present systems of assessment and learning activities did not clearly express which activities the students should be able to master at an independent level. The workshop exercises led to the elaboration of descriptions and limitations but no alteration of the 10 EPA titles.

The activities were vetted against the forthcoming national requirements for graduation. This exercise did not alter the phrasing or content. The vetting confirmed the importance of clarifying the level of supervision for clinical activities. The national requirements state that students shall be able to autonomously perform several professional tasks by the time they graduate.

The internal EQual validation was responded to anonymously by 15 participants from the working group. No EPA received a mean score below the recommended cut-off score 4.07 (Table 3). However, free text comments on details led to another round of minor clarifications of the descriptions and the anticipated required level of supervision.

**Table 3:** Results from the validation using the EQual rubric scores, 15 responders. For each EPA, 14 rubrics were scored by each respondent from 1–5, where 5 represented fulfilment. For each EPA the mean score for each responder and for the group was calculated, here presented with the mean and range. The group mean scores were also calculated separately for each of the three domains: discrete units of work = 6 items, entrustable, essential, and important tasks of the profession = 4 items, curricular role = 4 items

EPA	Total Mean (Range)		Discrete Activity Mean	Entrustable, essential and important Mean	Curricular role Mean
1	4.7	(3.9-5)	4.6	4.9	4.8
2	4.5	(3.4–4.9)	4.2	4.9	4.8
3	4.7	(4.1-5)	4.4	5.0	4.9
4	4.7	(3.8-5)	4.4	4.9	4.9
5	4.6	(3.6-5)	4.4	4.4	4.8
6	4.9	(4.5-5)	4.8	4.9	4.9
7	4.7	(4.1-5)	4.5	4.9	4.9
8	4.8	(4.4-5)	4.7	5.0	4.9
9	4.1	(2.9-5)	3.7	4.4	4.6
10	4.3	(3.2-5)	4.0	4.3	4.7

### Social validity

The survey was responded to, by 473 participants, of whom 461 stated that they were doctors, representing 31 different medical specialties. Time in practice was reported by 451 doctors: <5 years n = 52, 5–10 years n = 63, and > 10 years n = 337.

**Fig. 2:**
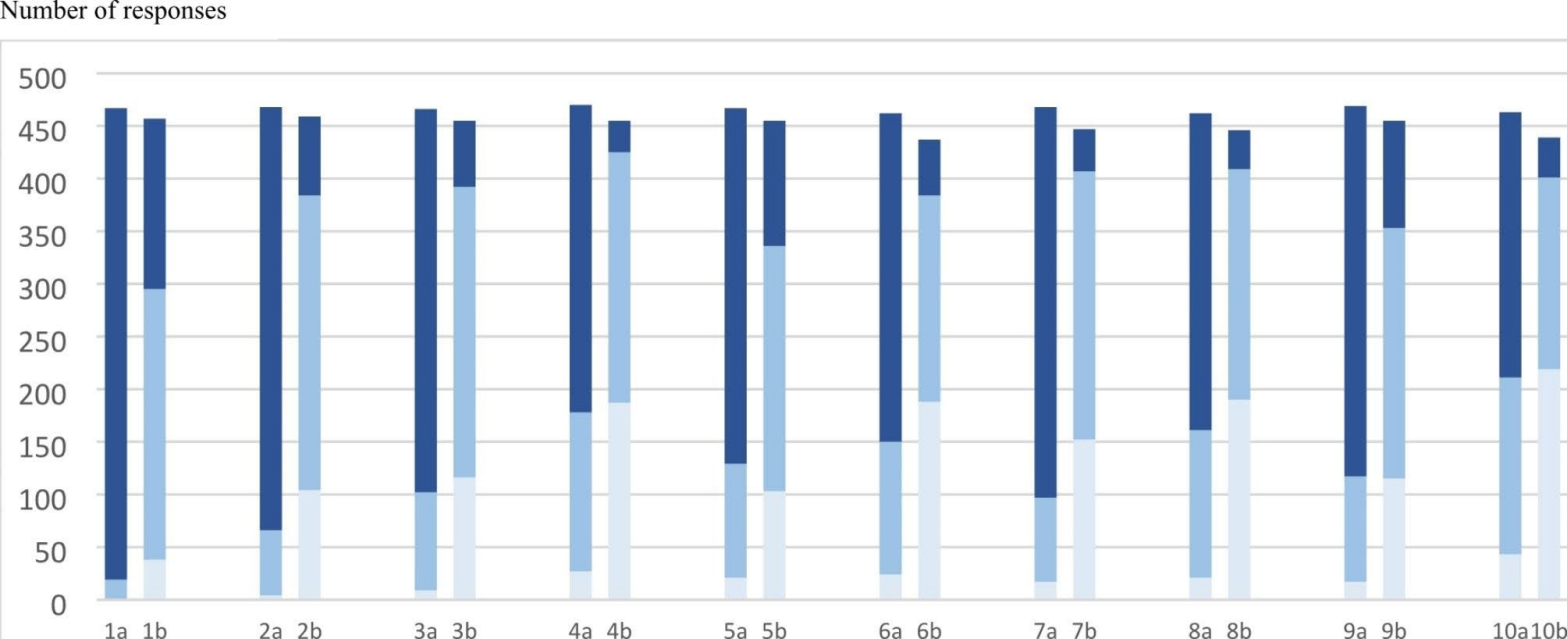
Responses to the questionnaire from all 473 participants, inquiring about the 10 EPAs regarding: **[a]** is the described activity common and of such importance that students should be able to perform it independently at the time for graduation (licensure)? (rated 1 = not important [light blue], 2 = somewhat important [medium blue], 3 = important [dark blue]). **[b]** how independent do you perceive graduates currently are? (rated 1 = not able at all [light blue], 2 = with supervision [medium blue], 3 = independent [dark blue]).

All activities were rated ‘important’ by 54%, or more, with EPA 1 reaching 96% (Fig. 2). The lowest ratings were found in EPA 10 (54%), EPA 4 (62%) and EPA 8 (65%).

When rating the current graduates’ independence, the performance of EPA 1 was rated as ‘independent’ by 35%, followed by EPA 5 (26%) and EPA 9 (22%). All other EPAs were rated ‘independent’ by less than 20%.

The results from the survey indicated a consistent pattern (Fig. 2). The responders found each activity important but noted a need for more practice to ensure independence in the new program.

**Fig. 3:**
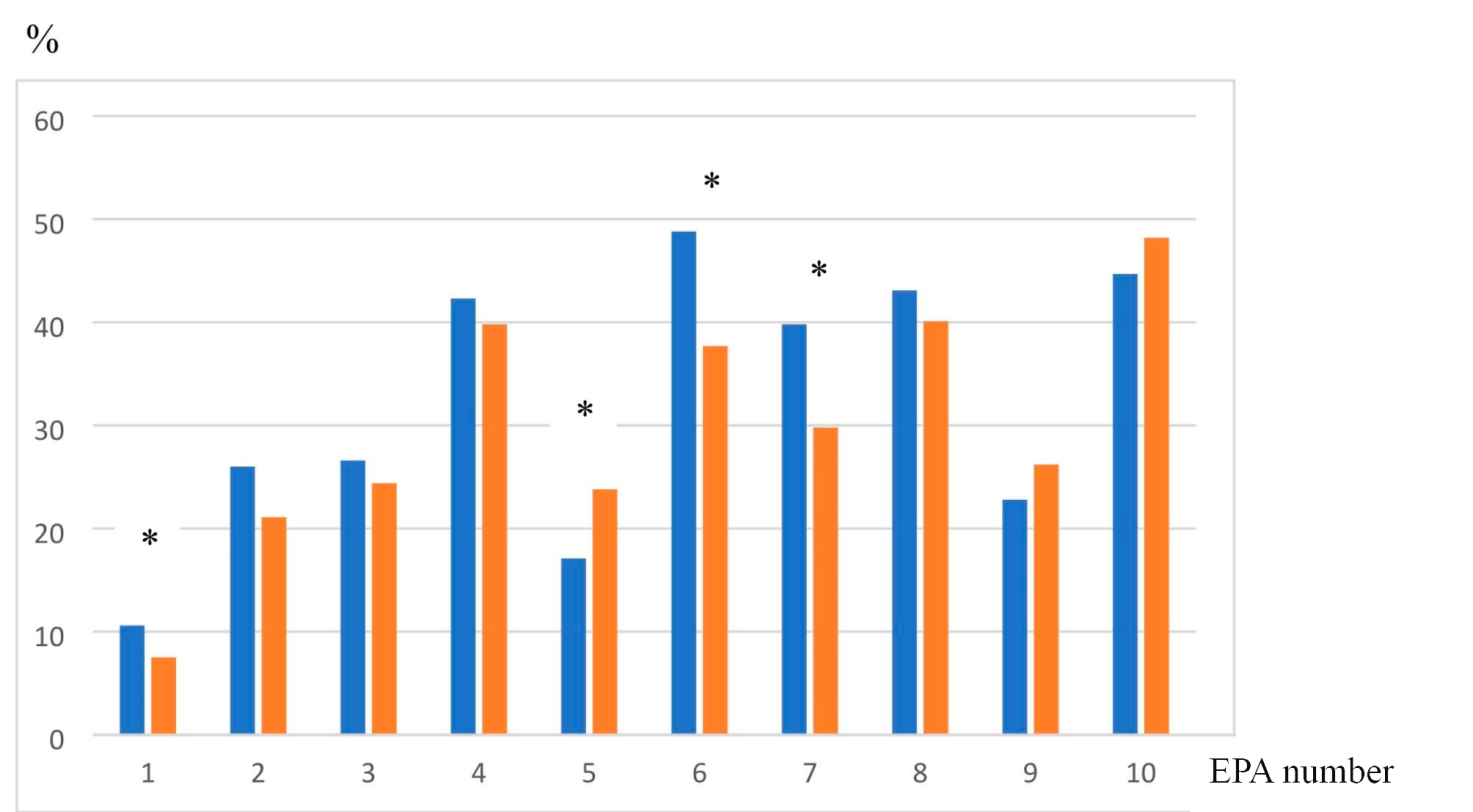
Responses from medical doctors, split into subgroups by time since graduation ≤ 10 years (blue, n = 115) and > 10 years (orange, n = 337). Proportion (%) of raters, selecting the option “not able” to perform EPA 1 through 10, when asked about the current graduates’ independence. *p < 0.05

The sub-analysis (Fig. 3) based on the responders’ time in practice (≤ 10 years and > 10 years), showed a significant difference between the groups selecting the option ‘not able’ (current graduates) for EPA 1, 5, 6 and 7 (p < 0.05).

A thematic analysis of the free text comments rendered three main themes with subthemes: Content within target areas [*Clinical authenticity, Speciality perspectives*], Definition of the activities [*Phrasing, Scope, Limitations*] and Clinical practice and learning [*Integrated practice, Feedback, Assessment]* (Table 4).

**Table 4:** Themes and sub-themes with examples of quotations from the content analysis of free text comment in the external survey

**Relevant target areas and content**	**Definition of the activities**	**Clinical practice and learning**
-Clinical authenticity *”After fullfillment of Internship (”AT”) you should be able to handle this, thus being a licensure requirement (EPA 7)* *”This is all a prerequisite for independent work as a medical doctor” (EPA 8)* - Speciality perspectives *”I miss inclusion of the ability to perform a clinical consultation/examination within the field of gynecology”* *“Clinical neurological examination”.* *[is missing]*	-Phrasing *“Strangely formulated heading with errors of referral”* - Scope *“The students can manage the classic disease presentations quite well. However, uncommon symptoms are more difficult and they can seldom formulate a complete investigation plan for a patient” (EPA 2)* - Limitations *“There is not enough focus directed towards the immediate action (EPA 7”)*	- Integrated practice *” I don’t know regarding adult health care but in pediatrics this is something that needs more training, thus requiring more educational hours”* *“It is already addressed but collaborative skills both regarding intra- and interprofessional practice can’t be emphasized enough”* *“ATLS needs to be included as mandatory”* - Feedback *” This of course calls for structured supervision during internship/corresponding clinical placements” (EPA 7)* *“ (EPA content)….does not reflect how strongly dependent newly graduated medical students have been, and are of bedside supervision)* - Assessment *“How to assess that the student can reason regarding a method’s reliability in relation to its properties and limitations?”* *“Difficult to assess in an adequate setting” (EPA 7)*

#### Content within target areas

Overall, the core EPAs were perceived as relevant and important, being fundamental for the work of a clinician. Some responders commented upon the lack of dedicated specialist focus within the EPA descriptions, e.g., dermatology, gynecology, and geriatrics.

#### Definition of activities

The phrasing of the EPAs was commented on, with suggestions to change single words or to relate to terms used in clinical practice. The scope of some EPAs was described as too extensive for undergraduate students when compared with activities during the current 5.5 years of undergraduate education. The EPAs considering a diagnosis and differential diagnoses, as well as a plan for further investigations, were by some regarded as comprehensive and theoretically heavy. Some respondents wanted clarifications on limitations within certain EPAs, especially regarding team management in the emergency room (EPA 7).

#### Clinical practice and learning

Comments related the EPAs to the new Swedish medical education, rendering a licensed MD, addressed the level of complexity, reflecting on the need for even more integrated clinical training and team-based professional practice, with the provision of clinical supervision and feedback. Concerns about the difficulties in assessing some of the EPAs prompted a focus on their implementation.

### Validation regarding acceptance by governmental stakeholders

Once the group had finalized the EPA descriptions and limitations, the framework was presented to and well received by representatives from national stakeholders (the National Board of Health and Welfare, the Swedish Society of Medicine, the Swedish Medical Association, the Swedish Association of Local Authorities and Regions, the Swedish Higher Education Authority, and the Department of Education).

## Discussion

We developed and began the validation of Core EPAs for the new Swedish 6-year undergraduate medical programs. The intent was to create a framework, relevant and easily understood by stakeholders. The work resulted in a national agreement on ten core professional activities that students should be entrusted to perform with a defined level of supervision at the time of graduation and licensing (Table 1).

The number of scientific articles focusing on the development of EPA and implementation strategies has increased in recent years. Several studies report high acceptability of the EPA model among teachers and clinical supervisors in undergraduate medical education, which contributes to its growing popularity [27, 28]. Our starting point was the Core EPAs reported by other countries. Overall, our EPA framework did not substantially differ from other frameworks, apart from the fact that we clarified that the activities were framed around a patient encounter. However, it was important to develop the framework so that it was aligned with the country-specific regulations and could not simply be translated from another context.

The Core EPAs should function as a framework for work-based learning and assessment. It was therefore prioritized to address aspects of validity in our context. In this phase of the development, there were no scores to interpret, but it was pertinent to ensure the high quality of the descriptions and that the descriptions were purposeful and socially accepted among medical doctors, including potential supervisors and future colleagues. The in-depth validation using EQual [11] confirmed that the EPAs were adequate. However, minor adjustments to the phrasing and the descriptions were made to improve clarity. At that time, the sub-scale analysis and cut-off values were not published, but it can be noted that the sub-scale analysis indicated a need for improvement, specifically for EPA 9 as a discrete task according to the suggested cut-off values for the sub-scales. [25] In the current version, EPA 9 is described as involving collaboration in a patient handover and patient discharge.

To guide the implementation phase, the exploration of social validity was an important step. In general, EPAs that aligned with more traditional medical school training (EPAs 1, 2, 3, 5, 7 and 9) were all rated as important (> 70% of the responders) and rather few responders (< 30%, 32% for EPA 7) thought that students were unable to conduct them with any sort of independence (Fig. 2). EPA 1 (Gather a history and perform a relevant physical examination) stood out as the most important activity (rated so by 92%) and the activity in which only a very few (8%) thought that the students were not independent at all at graduation. This was in agreement with the results of a study in the U.S. about the residents’ opinions on to what extent the different EPAs were practiced and assessed, where ‘history taking’ was the activity rated as the most occurring [29]. Even though in our study EPA 2, 3, 5, 7, and 9 were regarded as important (> 70%), 20–30% of respondents thought that the graduating students were not independent at all in these. This may indicate that the previous medical school curriculum would have needed further development regardless of any new legislation. This was further confirmed by the free text comments, where concerns regarding practice, feedback, and assessments were raised (Table 4).

The EPAs that demanded activities that earlier had not been the focus of Swedish medical schools (EPAs 4, 6, 8, and 10) were noted as important by fewer respondents (< 70%) and more respondents (> 30%) also thought that current graduates were not independent at all in the activities. EPAs 4, 6 and 8 all concern actual medical practice (i.e., practical healthcare), which has been, is, and will always be important for future physicians. These responses also suggest that, regardless of new legislation, the previous medical school curricula in Sweden may have needed further development. EPA 10 stood out, with only about 50% regarding it as important and about 50% thought that current students were not at all independent in the activity. From our perspective, patient safety (EPA 10) is very important and the foundation for all medical practice, and we have had difficulties finding valid arguments opposing this. The answers given probably reflect an unfamiliarity with patient safety in the context of medical schools, and this needs to be further explored during the EPA implementation. The activities included here were narrowed to identify a risk for a patient that could be observed in a patient encounter such as rounds or office visit (e.g., a care related infection, the risk of a pressure ulcer, or unneeded investigations). This was thus phrased to include risks that could be related to a patient encounter, in contrast to the wider definition used in the Core EPAs by the Association of American Medical Colleges (AAMC), where the EPA about safety also included system failures [9]. In a validity study based on the AAMC Core EPAs, the EPAs about performing procedures and contributing to a culture of safety were the ones where students had the lowest number of assessments registered [30]. This is consistent with the study by Ryan et al. [29] where the EPA about risks and system failures was rated as low in terms of the frequency of training and assessment.

We also wanted to explore if junior and senior doctors (defined as time in practice (≤ 10 years and > 10 years) perceived the lack of independence of the current graduates differently. In particular, the rating of ‘not at all’ was of interest as it could indicate areas where the learners are not allowed to practice. In the analysis no general conclusive differences were found. There was a significant difference found between the groups selecting the option ‘not able’ for EPA 1, 5, 6 and 7 (p < 0.05). However, for EPA 5 (Identify the need for and initiate interventions to promote health and prevent illness), the senior doctors more often perceived the graduates as not being able to perform the activity. On the contrary, for EPA 1, 6, and 7, the junior doctors rated ‘not at all’ significantly more often. The need to bridge the gap and enhance the transition between what the graduates are prepared to do and the needs for their next step has been addressed as one of the main desires by implementing EPAs for undergraduate medical students [31]. In a large national survey of recent graduates in the US [32], participants were asked to rate their own skills, for each of the 13 EPAs defined by AAMC. The three lowest rated were ‘Enter and discuss orders and prescriptions’, ‘Perform general procedures’ and ‘identify system failures and contribute to a culture of safety and improvement’. This emphasizes the importance of continuous monitoring of which EPAs students do get access to practice and what level of supervision is possible to reach during undergraduate studies.

Further efforts are needed to implement the new goals and EPAs in the curricula and make their necessity obvious for students, teachers, and supervisors. Within the Swedish curriculum, special attention may be needed to ensure that students do get sufficient training and feedback on the EPA 4, 6, 8, and 10. Students with experience from the use of EPA recommended early introduction to the Core EPAs and a model of shared responsibility for driving feedback [33]. These strategies, together with an infrastructure allowing the students to keep track of what they have practiced, may enable more opportunities to collect feedback on all EPAs.

The introduction of EPAs in medical education is not intended to replace frameworks that describe general knowledge, skills, and attitudes that a medical student needs to acquire. The medical program must still focus on the development of all the necessary knowledge, skills, and attitudes that are not suitable for the EPA framework but are required for the practice of the medical profession. It is important for the students and supervisors to understand the fundamental difference between an EPA and the complete set of knowledge, skills, and attitudes the individual must possess to be able to carry out the specific activity (EPA) independently [13]. To perform, e.g., EPA 4, ‘Formulate and implement an initial management plan’, requires knowledge of the disease and its treatment, the skills to analyze the situation and prioritize between different options, an ability to communicate with the patient, and an attitude that it is important to engage the patient in treatment decisions and respect the patient’s will. Assessing the student´s independence when performing an EPA is mere a way of gathering evidence for decisions about whether or not to entrust the student with the responsibilities of the activity at a specified level of supervision [13].

### Strengths and limitations

The setting of our work had several advantages. Firstly, teachers from all seven Swedish medical schools were represented, as were two students from the national student organization. Furthermore, the work was initiated and agreed on by national stakeholders for the medical schools, giving strong support for the process.

A limitation was that the members of the group were not experts on EPA when the process was initiated, but we strived to carefully review the literature and participate in international meetings to build our competence about the framework. It could also be seen as a limitation that we started by assessing frameworks from other nationalities as our starting point. However, as these frameworks were carefully developed, we believe they enabled our understanding of EPA as a concept. The frameworks were then critically appraised, both in view of our cultural context and our national requirements.

### Future steps

The next steps are the implementation at each medical school. The national collaboration will still be valuable to further explore aspects of validity as well as enablers and challenges in implementation. For the local implementation, there is a risk of underestimating the planning and resources needed, e.g. regarding supervisor training and infrastructure. Each school needs to design a specific program of assessments that includes adequate information for making entrustment decisions. For the implementation, important aspects will also include providing an infrastructure feasible for EPAs and entrustment decisions, including a digital system with learning analytics and feedback overviews to identify students’ progress and weaknesses. For the feedback/feedforward to be purposeful, supervisor training will be needed. It is then recommended that entrustment decisions be made by a committee [34] and for efficiency, collated data reports are thus needed. A purposeful digital infrastructure also enables a learner-driven approach to feedback and practice [35, 36], which is well aligned with a student-centered active learning approach. We also believe it would be valuable if the medical education for specialization continue to build on the EPA framework developed here, using the same vocabulary to facilitate the transition for the learners and supervisors.

## Data Availability

The datasets used and analyzed during the current study are available from the corresponding author on reasonable request.
